# The Impact of Internet Use on the Happiness of Chinese Civil Servants: A Mediation Analysis Based on Self-Rated Health

**DOI:** 10.3390/ijerph192013142

**Published:** 2022-10-12

**Authors:** Mengyuan Sui, Haifeng Ding, Bo Xu, Mingxing Zhou

**Affiliations:** School of Administration and Law, Hunan Agricultural University, Changsha 410100, China

**Keywords:** civil servants, Internet use, happiness, self-rated health

## Abstract

With the rapid socioeconomic development of China, studies related to Internet use and civil servants’ happiness have become a research hotspot in Chinese academia. This study empirically analysed the impact of Internet use on the happiness of Chinese civil servants using a sample of 3793 civil servants in Hunan Province, China. It showed that Internet use significantly enhanced the subjective well-being of Chinese civil servants. Furthermore, heterogeneity analysis revealed significant heterogeneity in the effect of the Internet on civil servants’ happiness, which varied across civil service groups with different education and gender. Moreover, the effect of Internet use on the happiness of the male and better educated civil servant groups was more pronounced than in the female and less educated civil servant groups. Additionally, mediation analysis revealed that Internet use and the happiness of civil servants were not linear, with health having a significant mediating effect. This indicates that Internet use helps civil servants maintain good health, and thereby enhances the happiness of civil servants. In addition, we also use a propensity score matching model (PSM) to address the endogeneity problem due to sample selectivity bias. The results show that the estimates are more robust after eliminating sample selectivity bias. The effect of Internet use on civil servants’ subjective well-being would be underestimated if the sample selectivity bias is not removed.

## 1. Introduction

Recently, the phenomenon of ‘civil service fever’ has severely affected the Chinese employment structure, which boasts approximately 7.2 million civil servants in China. As the socioeconomic environment continues to change, state governance also continues to improve its efficiency by reshaping the civil service; however, corresponding reform initiatives, such as the downward adjustment of salaries and benefits and the change in retirement pay to pension insurance [1], have reduced the social dominance of civil servants. Moreover, there is a more pronounced peer-to-peer competition at all levels of government in the process of improving governance effectiveness, which, in turn, evolves into pressure from higher levels to lower levels and intense competition between peers [2]. Thus, this imposes a great socioeconomic burden on public servants, resulting in lowered expectations of a better life and affecting the subjective well-being of the civil service community. As the core of the social system, the declining happiness of civil servants leads to decreasing job satisfaction, declining job performance and an increasing tendency to leave [1], which has serious social implications. Therefore, improving the happiness of the civil service group has currently become a key issue in social construction and development in China.

With the advent of the digital economy, the Internet has flourished in China, with the Internet penetrating every aspect of people’s lives and subtly changing traditional lifestyles, behavioural concepts and professional environments [3]. According to the 48th China Internet Development Statistics Report, as of June 2021, the number of Chinese Internet users has reached one billion and the Internet penetration rate has reached 71.6%. Indeed, the Internet has become an important and indispensable tool in modern life. Various studies have reported on its pivotal role in information transfer [4], economic development [5], social capital enhancement [6] and entertainment expansion [7]. Additionally, they also report on the relationship between the Internet and residents’ happiness [8]. However, existing studies only report on the happiness of residents in general, while that of the civil servants’ community remains lacking. Civil servants are managers who exercise the administrative authority of the state and perform the public affairs of the country. They also consist of the staff of the government administrative organs appointed under the legal procedures and methods, including grassroots government staff, legal service providers, social welfare department staff and other institutions [9]. In the new era, China is entering a new stage of development, the main social contradictions are changing and the people’s aspiration for a better life is growing, which requires accelerating the transformation of government functions and building a government governance system with clear responsibilities and administration in accordance with the law, which brings great pressure and challenges to the daily administrative work of public servants and affects the happiness of the civil service group. Enhancing the happiness of civil servants is conducive to motivating civil servants, improving their work efficiency and enhancing the credibility of the government. Furthermore, the Internet plays an important role in all aspects of civil servants’ lives, and it is of great practical significance to explore the impact of the Internet on the happiness of civil servants. Thus, this exploration not only aids in deepening our understanding of the Internet but also helps in utilising Internet tools to make civil servants happier. However, domestic and international studies on the impact of Internet use on civil servants’ happiness remain inconclusive. More micro-empirical results are needed to verify whether Internet use promotes the happiness of civil servants and whether different environments have an effect on the results. Methods to enhance the work ability of civil service groups and increase their work flexibility using the Internet, and thereby improve their happiness, have become an important and urgent practical issue. Based on the specific cultural environment in China, civil servants can generally be regarded as a whole group of people. Hunan is located in central China, in the middle reaches of the Yangtze River, and, as a rising province in the central region, civil servants in Hunan Province have strong typicality and representativeness in China. Accordingly, this study takes civil servants in Hunan Province as the research object and analyses the effect of Internet use on the happiness of civil servants to better understand the formation and evolution of civil servants’ happiness, to clarify the role of health in the relationship between Internet use and happiness, and enhance the happiness of civil servants.

The marginal contribution of this paper, unlike previous studies in this field, is, firstly, an empirical analysis of the impact of Internet use on the happiness of civil servants from the perspective of information technology. Second, this paper uses the Propensity Score Matching Model (PSM) to correct for the endogeneity problem caused by the selectivity bias of the civil service group and to ensure the robustness of the empirical results. Third, it aimed to explore health as an influencing mechanism between Internet use and happiness of civil servants, to verify its mediating effect, and to provide a new empirical basis for further clarifying the relationship between Internet use and happiness of civil servants. The study includes a literature review on happiness and the influence of the Internet on it. Additionally, it describes the main variables for descriptive statistics and constructs an econometric model.

## 2. Literature Review

According to the philosophy of hedonism, happiness is the ultimate goal of human beings. Happiness has various definitions; however, Ed Diner’s definition is generally accepted, which states that it is an individual’s subjective perception of the quality of their life according to individual criteria and is an important comprehensive indicator of the quality of one’s life [10]. Since the 1950s, happiness has been studied in Europe and the United States, mostly in the disciplines of psychology and sociology. In 1974, the American economist Easterlin proposed the famous ‘Easterlin paradox’. Since then, happiness has become a major research focus in the field of happiness economics. Throughout the years, studies on happiness have focused on the effects of economic factors on subjective well-being, such as income distribution [11], absolute income [12], relative income [13,14], unemployment rate [15], inflation [16], political factors, the degree of democratic development [17] and government public services [18]. Additionally, the effects of natural environmental factors (geography and air quality), leisure environmental factors [19,20], socio-demographic characteristics (age, gender, education, marital status and physical fitness) [21,22,23] and other individual factors (physical and psychosocial working conditions [24], psychological capital and self-esteem [25] and health [26]) on happiness have also been studied; happiness has been a goal of scholars. Improving people’s happiness has attracted extensive attention from scholars and many management practitioners in China and abroad. However, existing studies have mainly sampled college students [27], elderly people [28] or general residents [29] and very few civil servant groups.

The popularisation of the Internet has had a profound impact on the political, economic and cultural spheres, greatly impacting traditional lifestyles and modes of thinking. Studies have shown that the Internet can not only influence macro areas, such as industrial production, financial development and transportation [30,31], but also change residents’ consumption decisions, leisure and entertainment, political trust, democratic participation and moral values [32,33]. Accordingly, increasing studies have begun to focus on the impact of the Internet on well-being, reporting positive results. For example, Internet use positively impacts people’s happiness by providing information access, leisure and entertainment, interactive participation and online consumption [34]. The Internet not only provides people with new channels to access information, but also multiple modes of leisure and entertainment [35]. Moreover, the Internet, as a medium of social interaction, helps to increase the level of social interaction and enhance personal self-efficacy, thus reducing stress and depression levels [36]. However, the Internet may also have adverse effects. Long et al. reported that the use or lack of use of the Internet was not significant, whereas the frequency of Internet use was significant, which showed a significant increase in the happiness of the population [37]. Furthermore, it has also been suggested that excessive Internet use can replace daily interactions with family and friends, increasing the probability of isolation and creating negative emotions, and thereby reducing happiness [38]. Additionally, it has been speculated that the Internet is likely to increase loneliness and even induce psychological disorders, such as depression [39]. However, overall, studies suggest that the Internet enhances people’s happiness.

Health, as an important component of human capital, is both a prerequisite for the personal development of civil servants and a cornerstone of national development. The advent of the Internet has helped break down the knowledge barriers to health and helped the civil service community to better manage their health status. From the established studies, which focus mainly on residents, the elderly and young people, and less on civil servants, there are two broad views. For one thing, the use of the Internet contributes to health. It has been argued that the use of the Internet has contributed to an increase in the capacity of social healthcare services [40], especially e-health tools that help people to manage their health and access health information [41,42]. Studies have also analysed the impact of Internet use on mental health status and physical health status, with some suggesting that Internet use can improve social well-being and alleviate depression [43,44,45]. Hou’s study found positive effects of Internet use on people’s self-rated physical health, objective physical health and mental health [46]. Li believes that Internet use can help with health improvement and self-assessment of chronic disease conditions [47,48,49,50]. Secondly, the use of the Internet is not conducive to health [51]. Ning suggests that increased Internet use significantly increases sleep deprivation and obesity among rural adolescents, affecting their physical health [52].

**Figure 1:**
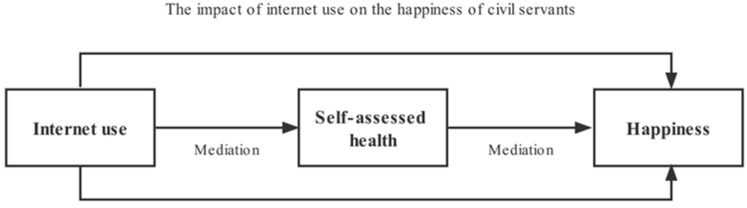
Theoretical framework diagram.

By sorting out the above literature, it is found that the literature on the use of the Internet and the sense of well-being in the academic circles ignores the civil servant group, and most of the current studies focus on the relationship between Internet use and health, or the factors influencing happiness; the research subjects are mostly residents, young people and the elderly, and there is a lack of research on civil servants. Therefore, this paper takes civil servants as the object of study and addresses the following questions: 1. Does Internet use have an impact on civil servants’ happiness? 2. If yes, are the results robust? 3. Do the effects of Internet use on the happiness of civil servants differ with different literacy and gender health status? 4. Does health influence the association between Internet use and the happiness of civil servants? The theoretical framework for this study is illustrated below (Figure 1).

## 3. Materials and Methods

### 3.1. Data Sources

Civil servants in Hunan Province, China, were focused upon as the survey target group. The survey covers 13 cities (states) and many departments, such as education, civil affairs, health, broadcasting, statistics, taxation and food and drug safety. The questionnaire survey was divided into two rounds and a total of 4000 questionnaires were distributed. The first round was from 1 October to 10 October 2021 and 1800 questionnaires were distributed; the second round was from 5 January to 10 February 2022 and 2200 questionnaires were distributed. A total of 3897 questionnaires were collected from both rounds, with a recovery rate of 97.4%. On excluding 104 invalid responses, 3793 valid questionnaires were finally selected. The survey sample comprised more men than women (58.4% men vs. 41.6% women), among which 43.5% were unmarried, 47.2 were married and 9.3% were divorced. In terms of education and income, 51.6% of civil servants had a bachelor’s degree and 30.5% had a master’s degree, while 69.4% had an annual income of less than CNY 100,000 and 25.7% had an income of CNY 100,000–200,000. Furthermore, the civil servants were mainly aged between 28 and 42 years (56.8%), indicating that most of the civil servants in the sample survey were young and strong, which is consistent with the basic structural requirements of civil servants.

### 3.2. Variable Design

#### 3.2.1. Dependent Variable

The core explanatory variable in the current study was happiness, and the happiness indicator is subjective feedback of an individual’s overall evaluation of the quality of life and their inner state [53]. Academic studies tend to consider subjective well-being to be the same as happiness, life satisfaction and quality of life, which are subjective indicators that can be evaluated effectively [54]. The whole questionnaire contains 35 questions in three sections. The personal information section, work situation, internet use and lifestyle sections were covered. Among them, happiness was measured through a five-point Likert scale. The questionnaire was designed to address the happiness of civil servants by asking, ‘Do you feel happy in your life?’ The options were ‘very unhappy’, ‘relatively unhappy’, ‘not happy’, ‘relatively happy’ and ‘very happy’. Furthermore, Cronbach’s alpha coefficient was calculated to be 0.895, indicating that the five measures have a high internal consistency. Accordingly, the mean values of the five indicators were calculated to constitute the ‘happiness’ variable of civil servants.

#### 3.2.2. Independent Variables

**Figure 2:**
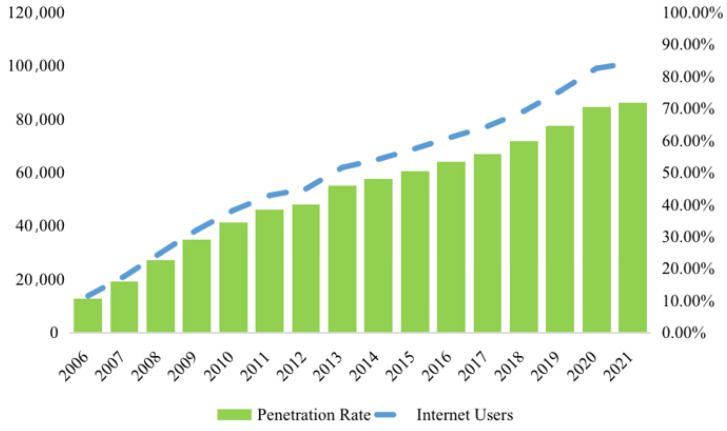
The development trend of the scale of Internet users in China from 2006 to 2021.

The development trend of the size of Chinese Internet users is as follows (Figure 2). Internet use is defined here as the act of civil servants in accessing Internet services via cell phones, computers and other terminal devices connected to communication networks, and it is the core explanatory variable of interest in this study. To ensure the accuracy of the research results, the questionnaire posed two questions, ‘Do you use a mobile device?’ and ‘Do you use a computer to surf the Internet?’, to measure Internet usage. If the respondent answered both questions online at the same time, a value of 1 was assigned; otherwise, a value of 0 was assigned.

#### 3.2.3. Mediating Variable

Mediation refers to the relationship between the mediating variable (M) that intervenes between the independent variable (X) and the dependent variable (X). In this study, the mediating variable was health, and the question ‘Are you currently healthy?’ was designed for this mediating variable. The response options were ‘healthy’ and ‘unhealthy’. Health is an important factor for civil servants to improve their happiness, and helping them improve their health through Internet technology is the key. Therefore, to fully understand the relationship between civil servants’ health, Internet use and happiness, this study represented ‘healthy’ and ‘unhealthy’ as 1 and 0, respectively, and included them in the regression model.

#### 3.2.4. Control Variables

Considering that civil servants’ happiness may also be influenced by other types of variables, this study ensured the robustness of the research results by introducing some control variables. Based on existing studies, controls for demographic variables were represented by gender, age, marital status and education level [21]; economic factors were represented by personal income [55]; and individual factors, such as smoking and drinking history, were included in the regression equation as control variables. The definitions and descriptive statistics of all variables are shown in Table 1. Additionally, for the absence of cointegration between variables, the VIF test was used. The results showed that all VIF values were less than 0.5, indicating that there was no multicollinearity problem. The VIF results are shown in Table 2.

### 3.3. Analysis Strategies

The research theme of this paper is the impact that technological advances have on the happiness of civil servants. The aim of the study is to explore the association between Internet use and the happiness of civil servants. Therefore, this paper uses the Order Probit model, Propensity Score Matching Model (PSM) and other methods to seek the relationship between the two. The dependent variable in this paper is happiness, which is a multi-categorical dummy variable with assigned values between 1 and 5, corresponding to the responses of ‘very happy’ and ‘happy’. In this paper, the ordered probit model was used to test the relationship between Internet use and civil servants’ happiness. The regression model was expressed as follows:

(1) $${\mathrm{Happiness}}_{i}^{*}={\;\alpha }_{1}+\mu_{1}{\mathrm{Internet}}_{i}+\mu_{1}X_{i}+\delta_{i}$$

In Equation (1), the ${\mathrm{Happiness}}_{i}^{*}$ is the first i latent variable of civil service happiness for the first survey respondent, representing the respondent’s i tendency to agree with the statement. The relationship of ${\mathrm{Happiness}}_{i}^{*}$ with ${\mathrm{Happiness}}_{i}$ is represented in the following relationship:

(2) $${\mathrm{Happiness}}_{i}=\{\begin{array}{c} 1,\;{\mathrm{Happiness}}_{i}^{*}\leq \;C1\;\\ 2,\;{C1<\mathrm{Happiness}}_{i}^{*}\leq \;C2\\ 3,\;{C2<\mathrm{Happiness}}_{i}^{*}\leq \;C3\\ 4,\;{C3<\mathrm{Happiness}}_{i}^{*}\leq \;C4\\ 5,\;{C4<\mathrm{Happiness}}_{i}^{*} \end{array}$$

(3) $$\begin{array}{c} {P(\mathrm{Happiness}}_{i}=1)=\phi \left(C1-X\beta \right)\\ {P(\mathrm{Happiness}}_{i}=2)=\phi (C2-X\beta )-\phi \left(C1-X\beta \right)\\ \cdots \cdots \end{array}$$

(4) $${P(\mathrm{Happiness}}_{i}=5)=1-\phi \left(C4-X\beta \right)$$

In Equations (2)–(4), C1, C2, C3 and C4 are the four critical points and, assuming that Pi(Y) is an individual i response of Y, the probability of Φ(−) is a standard normal cumulative distribution function. Our research procedures were questionnaire design, distribution, collection and data analysis. As the subject of our study was the relationship between Internet use and the happiness of civil servants, there were no ethical issues and all questionnaires were administered with the prior informed consent of the respondents. All research procedures conformed to the Helsinki requirements.

## 4. Results

### 4.1. Baseline Regression Results

The regression analysis (Table 3) shows that Internet use has a significantly positive effect on civil servants’ happiness. Furthermore, in model (1), the regression coefficient of the effect of Internet use on civil servants’ happiness is reported to be 0.085 and significant at the 1% level. In model (2), when the control variables of gender, age, marital status and education are added, the regression coefficient is observed to be 0.050 and significant at the 1% level. In model (3), the regression coefficient is reported to be 0.045 and significant at the 1% level when the control variables of job income, smoking history and drinking history were added. Therefore, the results indicate that Internet use is positively related to civil servants’ happiness.

For individual characteristics, gender showed a significantly negative effect on happiness in both males and females, with Internet use having a significantly positive association with happiness at the 5% level. In terms of marital status, unmarried status had a negative effect on happiness at the 5% level of significance, indicating that being married enhanced the happiness of civil servants, whereas divorce/widowhood had a significantly negative effect on the happiness of civil servants, indicating that divorce/widowhood is likely to decrease the happiness of civil servants. Regarding education, the happiness of civil servants with higher education was higher than that of civil servants with lower education at 10% significance, which can be attributed to the fact that higher education increases civil servants’ income, elevates their positions and, thus, enhances their happiness. Furthermore, civil servants who smoked had lower subjective well-being than non-smoking civil servants at the 10% significance level, which indicates that non-smoking civil servants tend to have a happier life, suffer less stress and have higher subjective well-being than civil servants who smoke regularly.

### 4.2. Robustness Test

To further analyse the robustness of the results, the Ologit model replacement measure for robustness testing was used. The regression analysis shows that Internet use on civil servants’ happiness was significantly positive at the 1% level, indicating that Internet use promotes civil servants’ happiness. The measurement results of model (2) and model (3) show that, after replacing the measures, married civil servants with low Internet use had lower levels of happiness. For civil servants with high literacy levels, the frequency of Internet use increased and the degree of happiness increased accordingly. These results are consistent with the above benchmark regression results, validating that the results have good robustness (Table 4).

### 4.3. Heterogeneity Analysis

The above results analysed the impact of Internet use on civil servants’ happiness and obtained their average effect. However, the results did not consider the differences in the happiness of different groups and the effect of these differences on Internet use. Therefore, the heterogeneity of the effect of Internet use on civil servants’ happiness in terms of literacy and gender was analysed here. Regarding education level, Internet use had a significant positive correlation with the happiness of civil servants with a master’s degree and above at the 10% level, and it positively affected the happiness of civil servants with a bachelor’s degree and below at the 10% significant level. This indicates that Internet use has a less significant happiness effect on civil servants with a higher education, whereas those with lower education are more inclined to use the Internet to facilitate work, leisure and entertainment. The reason for this is that, while highly educated civil servants may have higher incomes and status, highly educated civil servants may have higher expectations of the benefits of their education [56]. In terms of gender, a significant positive association between male and female Internet use on happiness was observed at the 1% level, with the happiness effect slightly higher in male civil servants than in female civil servants. This phenomenon can be attributed to the speculation that women are more delicate-minded and perceive happiness more strongly than men and, hence, are more likely to be affected by the negative effects of the Internet, leading to a lower level of happiness [57]. The estimated results are shown in Table 5.

### 4.4. PSM Model Eliminates Sample Selectivity Bias

The use of the Internet by different civil servants will be limited by related factors, such as personal characteristics. Therefore, the estimation results in this paper will suffer from endogeneity problems due to sample selection bias. In order to minimise the error of the estimation results, we use a propensity score matching model (PSM) to solve the endogeneity problem. There are many matching methods for propensity score matching. To ensure the robustness of the results, we select radius neighbour matching and kernel matching for analysis. According to the steps of PSM, it is necessary to carry out the sample balance test first. The validity of the estimated results can only be guaranteed by passing the sample balance test. The balance test results are shown in Table 6.

**Figure 3:**
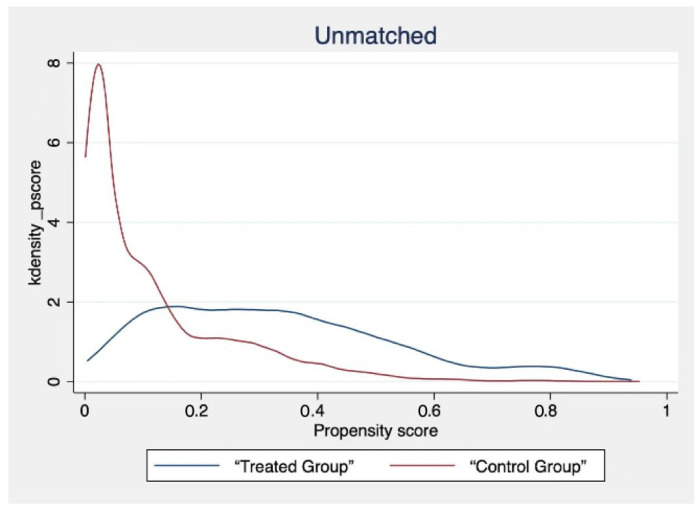
Kernel density function before matching.

**Figure 4:**
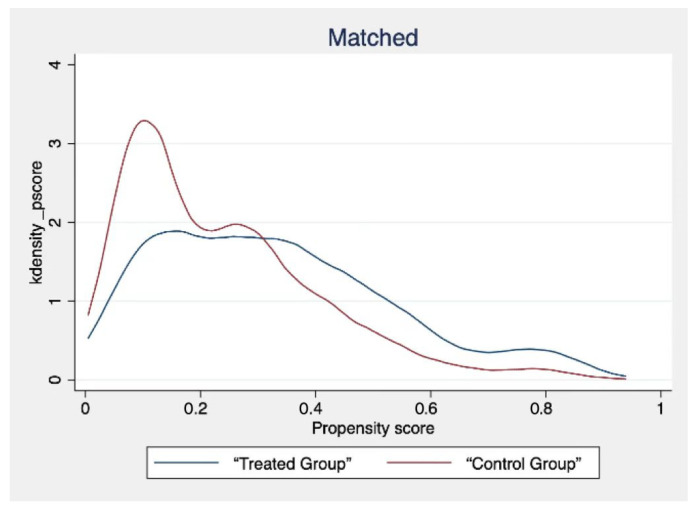
Kernel density function after matching.

According to the results in Table 6, the absolute values of all variable biases after matching are less than 5%. The *t*-test results showed that the *p* values of all variables were significant before matching but not after matching. This also shows that the PSM model has a good matching effect. In addition, in order to show the matching effect more intuitively, we also draw the kernel density function diagrams before and after matching (Figure 3 and Figure 4). According to the picture results, the matched kernel density function graphs tend to be more consistent. This shows that the PSM model effectively reduces the bias of the estimation results, and the obtained estimation results are purer.

Table 7 shows the analysis results of PSM. According to the results, the ATT value of the effect of Internet use on the happiness of civil servants was 0.028 when the endogenous problem of the PSM results was not used, and, after eliminating the endogenous problem, the ATT value became 0.033 and 0.035. This result suggests that the original estimates underestimated the impact of Internet use on the happiness of civil servants.

### 4.5. Internet Use and Civil Servants’ Well-Being: A Health Mediation Analysis

**Figure 5:**
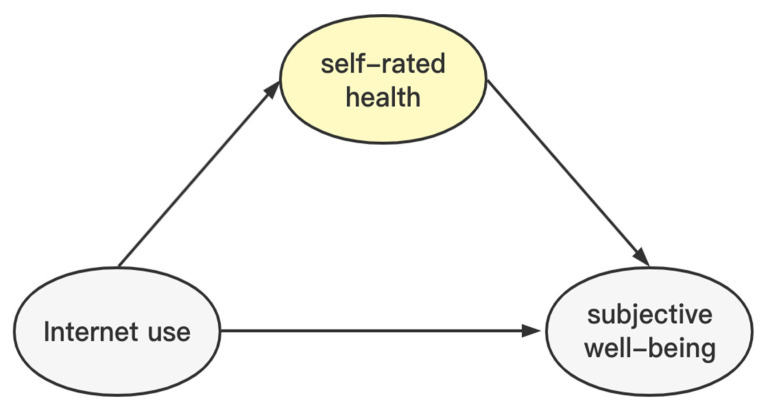
Mediation relationship.

The Internet not only brings great convenience to people’s life and work, but also completely changes people’s traditional life and behaviour patterns. To further clarify the influence of Internet use on civil servants’ happiness, this study considers civil servants’ health as a mediating variable and tests the channels of the effect of Internet use on the happiness of agricultural civil servants by constructing a mediating effect model (Figure 5). This study’s testing refers to Wen’s mediating effect factors [58], which include the behavioural basis of the influence mechanism of civil servants’ Internet use, to evaluate their influence on civil servants’ health.

1. To examine the effect of Internet use on the well-being of civil servants:
  (5) $${\;\mathrm{Happiness}}_{i}=\;\alpha +\mu_{1}{\mathrm{Internet}}_{i}+\mu_{1}X_{i}+\delta_{i}$$
2. Examining the impact of Internet use on the health of civil servants:
  (6) $${\;\mathrm{Healthy}}_{i}=\;\alpha +\mu_{1}{\mathrm{Internet}}_{i}+\mu_{1}X_{i}+\delta_{i}\;\;$$
3. Incorporating Internet use and health variables into the model simultaneously:
  (7) $${\mathrm{Happiness}}_{i}=\;\alpha +\mu_{1}{\mathrm{Internet}}_{i}+\mu_{1}{\mathrm{Healthy}}_{i}+\mu_{1}X_{i}+\delta_{i}$$
  where $X_{i}$ is the control variable and ${\;\mathrm{Healthy}}_{i}$ denotes the mediating variable. If the coefficient of Equation (4) is significant in the first step of the estimation results, it indicates that Internet use has a significant effect on civil servants’ happiness and allows for the continuation of the testing. Next, if the coefficient of Equation (5) is significant in the second step, it indicates that Internet use has a significant effect on the mediating variable health and allows for the third test to be conducted. In the last test step, health is added as a variable and, if the coefficient of the mediating variable in Equation (6) is significant and the Internet use variable is also significant, it indicates that there is a partial mediating effect of health between Internet use and civil servants’ happiness. However, if the coefficient of the mediating variable is significant and the Internet use variable is not significant, it indicates a full mediating effect (Table 8).

Table 8 shows that Internet use is significantly and positively related to civil servants’ happiness, i.e., Internet use has a facilitative effect on civil servants’ happiness and a significant positive effect on civil servants’ health. In the third step, both variables were included in the model, and it was found that this finding still holds. Moreover, health was found to play a significant mediating effect between Internet use and civil servants’ happiness, and the specific influence path is that Internet use enhances civil servants’ concern for their health, thereby enhancing civil servants’ happiness. Furthermore, with the development of the Internet, more and more civil servants have access to the Internet, wherein they can obtain information about a healthy lifestyle and health care, which, in turn, influences the physical exercise and habits of civil servants, gradually improving their health and, thus, enhancing their happiness.

## 5. Discussion

### 5.1. Summary of the Finding

This study shows that there is a significant difference between the happiness of civil servants who use the Internet and those who do not, with a significantly higher happiness level observed in those who use the Internet. Moreover, digital integration in the Internet era has increasingly become an important part of civil servants’ lives, and emerging information communication technologies, such as the Internet and cell phones, have a positive impact on civil servants’ happiness; this is consistent with the findings of Xu and Szabo [59,60]. This study shows the significant association of civil servants’ gender, marital status and education level with their Internet use and happiness. The happiness of female civil servants was observed to be higher than that of their male counterparts. This finding is consistent with the findings of Salavera et al. and Wang [61,62]. In traditional Chinese society, men are considered to be more stressed than women in both work and life, as men are traditionally given more responsibilities than women, thereby lowering their happiness. The happiness level of married civil servants was observed to be higher than that of unmarried civil servants. Furthermore, the higher the level of education of civil servants, the higher their happiness level, with more educated civil servants getting promoted faster and having higher salaries and, thereby, inducing a significant positive effect on happiness. Additionally, civil servants’ smoking history is significantly associated with their Internet use and happiness. Usually, civil servants relieve stress and feel happy by smoking; hence, it can be speculated that the happiness level of civil servants who smoke is lower than that of non-smoking civil servants. In summary, Internet use significantly enhances civil servants’ happiness, and the government should explore the potential of the Internet in improving workability and work flexibility by encouraging extensive participation in online learning and promoting the construction of digital labour platforms. Moreover, attention should be paid to guiding civil servants to use the Internet moderately in their daily lives, arranging their time online reasonably, paying attention to face-to-face interaction and communication and promoting family harmony. Guiding the ethos and orientation of the public’s online opinion, curbing exaggerated hype about wealth status and enhancing civil servants’ happiness through the Internet should be further looked into [48].

The results of the heterogeneity analysis showed that Internet use had a positive effect on civil servants with less than a bachelor’s degree, a master’s degree and above, and that the Internet had a slightly higher effect on civil servants with less than a bachelor’s degree than those with a master’s degree and above, which is in common with the findings of Luo and Liu [63] and Zhou [8]; at the same time, Internet use had a positive effect on both male and female civil servants, and the Internet had a slightly higher effect on men’s happiness than women’s, which is also in line with the studies of Lu et al. [64] and Zhu et al. [29]. Mediation analysis further showed that self-rated health partially mediated the relationship between Internet use and civil servants’ well-being; therefore, it indicated that Internet use affects civil servants’ self-rated health and, thus, their happiness. This study found that health has a significant positive correlation with both Internet use and happiness, with Internet use improving the health of civil servants and, subsequently, their happiness; this finding coincides with Ni and Pelaez-Fernandez [65,66]. This positive correlation can be attributed to the fact that information on health and medical care, online purchase of medications and other health-related queries are more easily accessible via the Internet, which influences their lifestyles, including eating habits and physical exercise. Nonetheless, this study confirms that health is a key factor affecting civil servants’ happiness. In general, civil servants who are physically active are more physically fit and have increased happiness. Therefore, medical technology enhancements can be promoted through digital technologies, such as the Internet, to guarantee medical services for civil service groups.

### 5.2. Policy Implication

This study has the following policy recommendations: first, to encourage civil servants to use the Internet and promote the application of Internet services to the civil service community, the government should explore more of the potential of the Internet to enhance work competency and work flexibility through initiatives such as encouraging widespread participation in online learning and promoting the development of digital labour platforms. Secondly, attention should be paid to guiding civil servants to use the Internet moderately in their lives, to arrange their time online reasonably, to attach importance to face-to-face interaction and communication, to promote family harmony, to guide the ethos and orientation of online public opinion, to curb over-exaggerated hype about wealth and status, and to give civil servants a greater sense of well-being in sharing the fruits of Internet development, so that the Internet can better benefit civil servants. Thirdly, medical technology can be promoted through digital technology, such as the Internet, to enhance medical services for the civil service community, and health knowledge and lifestyle can also be promoted through the Internet and mobile short videos to guide the civil service community to develop healthy habits.

### 5.3. Research Strengths and Limitations

This study has three strengths. Firstly, as China moves into a digital society, it is of great theoretical and practical importance to examine how digital integration, represented by the use of the Internet, can bring about changes to the working lives of civil servants and how to enhance the happiness of civil servants in the Internet era. Second, this study empirically analyses the impact of Internet use on civil servants’ happiness from an informational perspective, and uses a propensity score matching model (PSM) to correct for endogeneity problems caused by selectivity bias in the civil service population, ensuring the robustness of the model’s empirical results. Thirdly, this study explores health as an influential mechanism between Internet use and civil servants’ happiness and provides a new empirical basis for further clarifying the relationship between Internet use and civil servants’ well-being.

This study also has some limitations. First, due to the limited sample data, which evaluated the use of or lack of Internet use, this study could not present a comprehensive picture of the diversity of civil servants’ Internet use, such as the duration of Internet use, psychological motivation and type of content. According to relevant studies, appropriate Internet use can enhance happiness and health. However, excessive use may lead to Internet addiction, which may reduce happiness and health. Therefore, in the future, we will further explore the impact of the frequency of Internet use on the happiness of civil servants, so as to reveal the link between technological advancement and happiness in a more scientific way. Second, the data in this paper are mostly the subjective evaluations of the respondents, who may have concealed their actual self-evaluation. Future studies should introduce a longitudinal design or experimental design to test this. Additionally, this study did not specifically divide the mediating variable of health and used a self-rated health measure. However, the mental health of civil servants is also increasingly becoming a focus of social concern. Therefore, in the future, we will explore the relationship between internet use on civil servants’ health by dividing health along several dimensions.

## 6. Conclusions

This study revealed that Internet use can significantly enhance the happiness of Chinese civil servants. Furthermore, civil servants who use the Internet have higher happiness levels than those who do not use the Internet. Additionally, heterogeneity analysis revealed that the effect of the Internet on civil servants’ happiness significantly varied across civil service groups based on education and gender, with Internet use having a more pronounced effect on the happiness of male and more educated civil service groups than female and less educated civil servants. Furthermore, health was reported to play a significant mediating role between Internet use and happiness, suggesting that Internet use helps civil servants maintain good health and, thus, enhances their happiness. In addition, we also use a propensity score matching model (PSM) to address the endogeneity problem due to sample selectivity bias. The results show that the estimates are more robust after eliminating sample selectivity bias. The effect of Internet use on civil servants’ happiness would be underestimated if the sample selectivity bias is not removed.

## Figures and Tables

**Table 1:** Descriptive statistics of the variables.

Variable Name	Variable Definition	OB	Mean	SD
Dependent Variable				
Civil Service Happiness	1–5, the higher the score, the higher the happiness	3793	3.541	0.957
Independent Variable				
Internet Usage	No = 0, Yes = 1	3793	0.936	0.244
Control Variables				
Gender	Female = 0, Male = 1	3793	0.366	0.482
Age	Continuous Variable (years)	3793	32.644	5.995
Marital Status	Unmarried = 1, Married = 2, Divorced = 3	3793	1.858	0.578
Education Level	College and below = 1, Bachelor = 2, Master = 3, Doctor = 4	3793	2.443	0.679
Job Income	Continuous Variables.	3793	50,294.53	46,293.55
Smoking History	No = 0, Yes = 1	3793	0.086	0.280
Drinking History	No = 0, Yes = 1	3793	0.057	0.232

**Table 2:** Multicollinearity Test.

Variable	VIF
Gender	1.56
Age	1.83
Marital Status	1.72
Education Level	1.34
Job Income	1.66
Smoking History	1.52
Self-assessment of Health	1.28

**Table 3:** Baseline regression results of Internet use on civil servants’ well-being.

Variables	Model (1)	Model (2)	Model (3)
	Happiness	Happiness	Happiness
Internet Usage	0.085 *** (0.035)	0.050 *** (0.041)	0.045 *** (0.042)
Gender		−0.011 ** (0.036)	−0.010 ** (0.041)
Age		0.001 (0.008)	0.001 (0.003)
Marital Status		−0.008 ** (0.033)	−0.009 ** (0.033)
Education Level		0056 ** (0.031)	0.050 ** (0.032)
Job Income			0.000 (0.000)
Smoking History			−0.128 * (0.066)
Drinking History			0.019 (0.077)
Sample Size	3793	3793	3793
Adj-R2	0.0006	0.0009	0.0014

Note: *, ** and *** indicate significance at 10%, 5% and 1%, respectively.

**Table 4:** Robustness test results of Internet use on civil servants’ happiness.

Variables	Model (1)	Model (2)	Model (3)
	Happiness	Happiness	Happiness
Internet Usage	0.097 *** (0.031)	0.053 *** (0.041)	0.046 *** (0.042)
Gender		−0.016 ** (0.033)	−0.010 ** (0.041)
Age		0.001 (0.003)	0.001 (0.003)
Marital Status		−0.007 ** (0.030)	−0.007 ** (0.029)
Education Level		0069 ** (0.028)	0.061 ** (0.029)
Job Income			0.000 (0.000)
Smoking History			−0.118 ** (0.060)
Drinking History			0.010 (0.070)
Sample Size	3793	3793	3793
Adj-R2	0.002	0.003	0.003

Note: ** and *** indicate significance at, 5% and 1%, respectively.

**Table 5:** Heterogeneity test for different literacy levels and different gender status.

Variables	Education Level		Gender	
	Undergraduate and Below	Master’s and Above	Women	Male
	Civil Service Happiness		Civil Service Happiness	
Internet Use	0.070 ** (0.038)	0.617 * (0.369)	0.047 * (0.052)	0.053 * (0.071)
Control Variables	Control	Control	Control	Control
Adj-R2	0.0011	0.0382	0.0044	0.0033

Note: * and ** indicate significance at, 10% and 5%, respectively.

**Table 6:** Sample matching quality balance test.

Variables	Unmatched Matched	Mean		Bias (%)	Reduce Bias (%)	*t*-Test	
		Treated	Control			*t*-Value	*p* > |*t*|
Gender	Unmatched	0.258	0.183	28.6	98.3	7.02	0.000
	Matched	0.258	0.278	−1.3		−0.13	0.965
Age	Unmatched	38.345	45.331	−67.2	99.2	−20.30	0.000
	Matched	38.345	48.945	1.0		−0.78	0.520
Marriage status	Unmatched	1.826	1.322	−32.1	95.1	−10.13	0.000
	Matched	1.826	1.190	0.7		−0.60	0.676
Education level	Unmatched	2.712	2.603	11.8	94.0	30.28	0.000
	Matched	2.712	2.776	2.0		5.40	0.112
Job Income	Unmatched	50,378.12	49,331.02	10.4	91.1	13.09	0.003
	Matched	50,378.12	50,213.07	1.9		0.35	0.875
Smoking History	Unmatched	0.092	0.078	11.2	99.5	6.12	0.000
	Matched	0.092	0.101	3.0		0.38	0.812
Drinking History	Unmatched	0.067	0.012	56.0	93.4	19.13	0.000
	Matched	0.067	0.109	3.6		0.77	0.529

**Table 7:** Results of PSM estimation.

	Subjective Well-Being			
	Treated	Control	ATT	SE
Unmatched	2.389	2.435	0.028	0.024
Matched				
Radius neighbour matching	2.385	3.429	0.033	0.028
kernel matching	2.380	3.423	0.035	0.028

**Table 8:** Estimated results of mediating effects of self-rated health.

Steps	Civil Service Happiness		
	Step 1	Step 2	Step 3
Dependent Variable	Happiness	Self-rated Health	Happiness
Internet Use	0.045 ** (0.042)	0.130 *** (0.087)	0.052 ** (0.042)
Self-rated Health			0.495 *** (0.078)
Control Variables	Control	Control	Control
Adj-R2	0.0139	0.0376	0.0134
Sample Size	3793	3793	3793

Note: ** and *** indicate significance at, 5% and 1%, respectively.

## Data Availability

Not applicable.
